# Testing the Predictive Validity of the Healthy Eating Index-2015 in the Multiethnic Cohort: Is the Score Associated with a Reduced Risk of All-Cause and Cause-Specific Mortality?

**DOI:** 10.3390/nu10040452

**Published:** 2018-04-05

**Authors:** Chloe E. Panizza, Yurii B. Shvetsov, Brook E. Harmon, Lynne R. Wilkens, Loic Le Marchand, Christopher Haiman, Jill Reedy, Carol J. Boushey

**Affiliations:** 1 University of Hawaii Cancer Center, Honolulu, HI 96813, USA; CPanizza@cc.hawaii.edu (C.E.P.); YShvetso@cc.hawaii.edu (Y.B.S.); Lynne@cc.hawaii.edu (L.R.W.); Loic@cc.hawaii.edu (L.L.M.); 2 University of Memphis, Memphis, TN 38152, USA; bharmon1@memphis.edu; 3 University of Southern California Norris Comprehensive Cancer Center, Los Angeles, CA 90033, USA; Christopher.Haiman@med.usc.edu; 4 Division of Cancer Control and Population Sciences, Bethesda, MD 20892, USA; reedyj@mail.nih.gov

**Keywords:** Healthy Eating Index, Dietary Guidelines for Americans, dietary index, multiethnic, mortality risk, Dietary Practices Methods Project, prospective cohort study

## Abstract

The Healthy Eating Index-2015 (HEI-2015) was created to assess conformance of dietary intake with the Dietary Guidelines for Americans (DGA) 2015–2020. We assessed the association between the HEI-2015 and mortality from all-cause, cardiovascular disease (CVD), and cancer in the Multiethnic Cohort (MEC). White, African American, Native Hawaiian, Japanese American, and Latino adults (*n* > 215,000) from Hawaii and California completed a quantitative food-frequency questionnaire at study enrollment. HEI-2015 scores were divided into quintiles for men and women. Radar graphs were used to demonstrate how dietary components contributed to HEI-2015 scores. Mortality was documented over 17–22 years of follow-up. Hazard ratios (HRs) and 95% confidence intervals (CIs) were computed using Cox proportional hazards models. High HEI-2015 scores were inversely associated with risk of mortality from all-cause, CVD, and cancer for men and women (*p*-trend <0.0001 for all models). For men, the HRs (CIs) for all-cause, CVD, and cancer comparing the highest to the lowest quintile were 0.79 (0.76, 0.82), 0.76 (0.71, 0.82), and 0.80 (0.75, 0.87), respectively. For women, the HRs were 0.79 (0.76, 0.82), 0.75 (0.70, 0.81), and 0.84 (0.78, 0.91), respectively. These results, in a multiethnic population, demonstrate that following a diet aligned with the DGAs 2015–2020 recommendations is associated with lower risk of mortality from all-cause, CVD, and cancer.

## 1. Introduction

Dietary pattern analysis is a relatively new method to quantify diet quality, monitor changes in population-based diet quality, and assess the relationships between diet quality and health-related outcomes [1,2,3,4]. Analyses of individual nutrients and foods are still important; however, as we consume foods in combination, the synergistic effect of food on health must be considered [2]. Studying diet patterns also allows researchers to promote dietary recommendations in the form of patterns, which may be easier for the general public to interpret and follow [5]. 

Dietary indices are important tools used to assess dietary patterns. They may be derived from theoretically based scoring systems or a priori approaches, which are guided by evidence-based research [2]. For example, the Healthy Eating Index (HEI) is constructed to reflect the evidence-based recommendations of the Dietary Guidelines for Americans (DGA) and to evaluate conformance to these recommendations [6,7,8,9]. Every five years the Dietary Guidelines Advisory Committee reviews current nutrition research and provides an Advisory Report to the United States Department of Agriculture (USDA) and Health and Human Services (HHS) [4,10]. Guided by this report, the USDA and HHS publish the Dietary Guidelines for Americans (DGA), which provide evidence-based food and beverage recommendations to the public [4,10]. Since the implementation of the original HEI in 1995, development and application of successive HEIs to assess and monitor dietary status have been ongoing [3,6,11]. The HEI-2005 introduced density-based scoring standards, which were continued in the HEI-2010 and HEI-2015. Additional changes to scoring standards were implemented between each new HEI to reflect updates in the DGA [3,12,13].

To add to the evidence for using dietary patterns to guide dietary advice, the National Cancer Institute (NCI) initiated the Dietary Patterns Methods Project (DPMP) [13,14,15]. This is a collaborative project between three large cohort studies, including the NIH-AARP Diet and Health Study (AARP) [16,17], the Multiethnic Cohort Study (MEC) [18,19] and the Women’s Health Initiative Observational Study (WHI-OS) [20,21]. These groups adopted overlapping and complementary research questions and conducted standardized diet analysis using four dietary indices, including the HEI-2010, the Alternative Healthy Eating Index-2010 (AHEI-2010), the alternate Mediterranean Diet (aMED), and the Dietary Approaches to Stop Hypertension (DASH) [15]. A summary of the results revealed that men and women with higher quality diets (higher index scores) were at significantly lower risk (11–28%) of death from all-causes, cardiovascular disease (CVD), and cancer compared to people with lower quality diets (lower index scores) [15]. This was true for mortality assessments made with each dietary index, with the exception of women from the WHI-OS cohort, where the AHEI-2010 score was not associated with cancer death [15].

The HEI-2015 was introduced to reflect the DGA 2015–2020 [12]. The HEI-2015 is comprised of 13 components and the maximum HEI score is 100 points [12]. There are nine adequacy components (foods to eat enough of) including Total Fruits, Whole Fruits, Total Vegetables, Greens and Beans, Whole Grains, Dairy, Total Protein Foods, Seafood and Plant Proteins, and Fatty Acids. There are four moderation components (foods to limit) including Refined Grains, Sodium, Added Sugars, and Saturated Fats. Most components are scored on a density basis, that is, amounts per 1000 kcal of intake [12]. The Fatty Acids component is scored using the ratio of poly- and mono-unsaturated fatty acids (PUFAs and MUFAs) to saturated fatty acids (SFAs), and Added Sugars and Saturated Fats are scored as a percentage of total energy intake. The key differences between the HEI-2010 and the HEI-2015 include the scoring for legumes and the Empty Calories component. In the HEI-2010, legumes were allocated into two components and have expanded into four components with the HEI-2015, including Total Vegetables, Greens and Beans, Total Protein Foods, and Seafood and Plant Proteins [12]. In the HEI-2015, the Empty Calories single component was replaced with the two components of Saturated Fat and Added Sugars [12]. Alcohol is no longer included in the Empty Calories component score; instead, the energy (kcal) from alcohol is now added to the total energy intake per day [12]. 

To assess the efficacy of the new HEI score, the AARP, MEC, and the WHI-OS each completed a standardized mortality assessment using the HEI-2015. The aim of this study is to examine the association between the HEI-2015 and mortality from all-cause, CVD, and cancer in the MEC, which represents a large cohort of adult men and women from five distinct ethnic groups residing in Hawaii and Los Angeles (LA).

## 2. Methods

### 2.1. Study Population

The MEC is a large prospective cohort study developed to investigate the relationship between diet and health-related outcomes among African American, Latino, Japanese American, Native Hawaiian, and white men and women [18]. Hawaii and the LA basin have large multiethnic populations; therefore, participants were recruited from these areas between 1993 and 1996. A detailed report of the MEC study design and implementation has previously been published [18]. Briefly, inclusion criteria were men or women, 45–75 years and living in Hawaii or LA at cohort entry. Driver’s license files were the primary resource used to identify potential participants. Secondary resources included voter registration and Medicare files. The final sample included over 215,000 individuals, who identified as African-American (16.2%), Latino (21.2%), Japanese-American (26.4%), Native Hawaiian (6.7%), white (23%) or other ancestry (6.5%). Socio-demographic, anthropometric, health history, physical activity and dietary intake information were collected at cohort entry using a 26-page self-administered questionnaire (Qx1). The institutional review boards at the University of Hawaii and the University of Southern California approved the study.

### 2.2. Dietary Assessment and Calculation of the Healthy Eating Index-2015 (HEI-2015)

All participants were provided with the Qx1 written in English, and Latinos were provided English and Spanish language versions [18]. A detailed description of the development and validation of the Qx1 is published elsewhere [18,22]. The Qx1 included a quantitative food-frequency questionnaire (QFFQ), developed to be suitable for the five main ethnic groups in the study [18]. The QFFQ was validated and calibrated in each ethnic-sex group [22]. Dietary intake over the last year was assessed using 182 questions. Questions were grouped into key categories, e.g., meats, bread items, and alcoholic and other beverages. The frequency of consumption was assessed using ordinal categories ranging from “never or hardly ever” to “4 or more times a day”. Serving sizes were also assessed using categories with the format varying by food. Images of different portion sizes were provided to help participants estimate the serving size of some food items, e.g., stir-fried beef. For other food items, three different written serving size options were provided without images, e.g., ½ hot dog, 1 hot dog, 2 hot dogs or more. A customized multiethnic food composition database was developed for the MEC [18,19,23]. This database was used to calculate food groups according to the MyPyramid Equivalents Database (MPED) [24,25]. The MPED is a system developed by the USDA to help researchers examine dietary data in terms of MyPyramid food groups (e.g., total vegetables, added sugars). Dietary intakes using the MyPyramid food groups were calculated from the QFFQ by summing each participant’s daily servings across the food items. The HEI-2015 scores were calculated using the MPED group and subgroup data [24]. SAS program code to calculate HEI-2015 scores was created specifically for the DPMP by the NCI to insure harmonization in coding across the cohorts participating in the DPMP project [14].

### 2.3. Healthy Eating Index-2015 (HEI-2015) Scoring

The HEI provides a score for diet quality and not diet quantity. For example, the index can provide information on the quality of the food consumed, but not whether an individual is meeting his or her nutrient requirements [13]. A quality versus quantity approach was chosen because it allows for the comparison of scores between age and sex groups [13]. An overview of HEI-2015 components and scoring standards can be found in Table 1. The HEI-2015 aligns with and reflects the changes between the 2010 DGA and 2015–2020 DGA [12]. The HEI-2010 and HEI-2015 have the following features in common: adequacy and moderation components, the same adequacy components, most components scored on a density basis, and standards for recommendations based on the least restrictive standards, e.g., least restrictive sodium recommendations [12]. As previously mentioned, scoring standards for legumes and the Empty Calories component have changed. Also, energy from alcohol consumed is now added to total energy intake per day for HEI-2015, which is used as the denominator for density values. This change results in a lower HEI-2015 score for an individual who consumes alcohol compared to an individual with the same diet who does not. For example, for the Total Fruits component, computed as (Total Fruits in cup equivalents per day/energy intake per day) × 1000 kcal) [13], will have a larger denominator for the individual who consumes alcohol, thereby decreasing the Total Fruits component score and HEI-2015 score. 

To achieve the maximum HEI-2015 score of 100 points, an individual would need to score maximum points on all components. For each component, intakes closer to the DGA recommendations increase the score. Therefore, to achieve a perfect HEI-2015 score, individuals need to have a high intake of foods that count toward the adequacy components and a low intake of foods that count toward moderation components (moderation components are scored inversely). Scores between the minimum and maximum standards for each component are computed proportionately [3].

### 2.4. Case Ascertainment

Deaths were identified by using state death files and the National Death Index. Deaths from CVD were identified and classified as International Classification of Diseases, Ninth Revision (ICD9) codes 390–448 or International Classification of Diseases, Tenth Revision (ICD10) codes I00–I78 and G45 [26,27]. Cancer deaths were identified by using ICD9 codes 140–209 or ICD10 codes C00–C96 [26,27]. All-cause mortality included CVD and cancer deaths as well as deaths from other causes, including accidents and suicides. All death files were current as the closure dates of 31 July 2015 for participants in Hawaii and 31 March 2015 for participants in LA. Participants with no recorded deaths as of these closure dates were censored.

### 2.5. Statistical Analysis

Analyses were limited to 70,170 men and 86,634 women who identified with one of the five main MEC ethnic groups (white, African American, Japanese American, Native Hawaiian, and Latino) (excluding *n* = 13,992), had valid dietary assessment information (excluding *n* = 8263); and had no previous history of cancer, heart attack, or stroke at baseline (excluding *n* = 36,723). The association of all-cause, CVD, and cancer mortality with HEI-2015 was modeled separately for men and women through Cox regression using years since study entry as the time metric. Cox regression was run for all ethnic groups combined and for each ethnic group separately. To assess if any one particular component was driving the association of HEI-2015 with mortality outcomes, we examined separate Cox models for HEI-2015, removing one dietary component at a time [16]. The association of mortality outcomes with the HEI-2015 was also modelled on the MEC sample with 17,012 fewer mortality cases from the follow-up period of the HEI-2010 mortality analysis [19]. Sensitivity analysis with the same number of cases as the previous study [19] was performed to compare the difference between HEI-2010 and HEI-2015, and so that any observed differences would not be influenced based on the additional mortality cases. For CVD and cancer models, study participants who died of other causes were censored at the time of death. The following self-reported covariates were included in all models: age at study entry and energy intake as continuous variables, history of diabetes (yes or no), ethnicity (as indicator variables), weekly hours of moderate-to-vigorous physical activity (<2.5 or ≥2.5 h/week), smoking (current smoker, past smoker, or never smoked), education (<12, 12, 13–15, or ≥16 years) as a proxy of socioeconomic status, marital status (married or not married), and hormone-replacement therapy (HRT) (yes or no (women only)). BMI (in kg/m^2^) was categorized as ≤24.9, 25–29.9, or ≥30 using self-reported height and weight. The HEI-2015 does not have a unique component for alcohol; therefore, models were further adjusted for alcohol intake in the past year as a continuous variable. All variables included as continuous measures had no missing values. Education, marital status, smoking, BMI, and weekly hours of moderate to vigorous physical activity had missing values; thus, each of these variables were modeled with a separate missing value category. Missing values ranged from <1% to 2.3% of the total sample. Separate models were fit for men and women with all ethnic groups combined. Total HEI-2015 scores and HEI-2015 with one component removed were divided into quintiles. HRs and 95% confidence intervals (CIs) were calculated for each quintile using the lowest quintile as a reference category. Wald’s chi square statistic was used to evaluate the linear trend on the basis of the median dietary score within each quintile. The proportional hazards assumption for Cox models was verified by plotting scaled Schoenfeld’s residuals against the time to the event [28]. Mean HEI-2015 scores by ethnic group and sex were compared using ANOVA. Given the ANOVA results were statistically significant for both men and women, post hoc analysis was undertaken using Scheffe’s multiple comparisons procedure.

Radar graphs were constructed to provide a visual representation of how men and women in quintile 1 and quintile 5 obtained their overall HEI-2015 scores [29]. Each axis on a radar graph represents a unique component score. Component scores were graphed as percentages, e.g., a Total Fruits score of 4/5 was graphed as 80%. A perfect HEI-2015 score (100% for each component) would be displayed as a line around the border of the radar graph. All descriptive analyses were conducted with IBM SPSS Statistics version 23 software (IBM Corp., Armonk, NY, USA), all statistical modeling was conducted with SAS version 9.4 software (SAS Institute Inc., Cary, NC, USA), and radar graphs were created using Microsoft Excel 2013 (Microsoft Corp, Redmond, WA, USA). All *p* values were 2-sided, and *p* < 0.05 was defined as statistically significant.

## 3. Results

### 3.1. Participant Characteristics 

A total of 51,442 mortality cases were documented (26,376 men and 25,066 women) over 17–22 years of follow-up (Table 2). Of these cases, 17,662 deaths were from CVD (9130 men and 8532 women) and 14,778 were from cancer (7812 men and 6966 women). Compared to men and women in quintile 1 (lowest diet quality), participants in quintile 5 (highest diet quality) on average were older at the time they completed the Qx1, had a lower BMI, lower energy intake, and more weekly hours of moderate to vigorous physical activity. Quintile 5 also contained a larger proportion of people with diabetes, those who never smoked, and those who graduated from college, compared to quintile 1. Among both men and women, there were higher proportions of Japanese American, Latino, and Native Hawaiian participants in quintile 1 compared to quintile 5, and a higher proportion of white and African American participants in quintile 5 compared to quintile 1. There was a lower proportion of married women in quintile 5 than in quintile 1, and a higher proportion of married men in quintile 5 than in quintile 1. A higher proportion of women in quintile 5 were users of hormone-replacement therapy, compared to all other quintiles. Mean HEI-2015 scores by sex and ethnic group are in Appendix A, Table A1.

### 3.2. Mortality Analysis

For men and women, participants in quintile 5 were at lower risk of all-cause, CVD, and cancer mortality compared to participants in quintile 1 (Table 3). For men, the quintile 1: quintile 5 HRs (95% CIs) for all-cause, CVD, and cancer for were 0.79 (0.76, 0.82), 0.76 (0.71, 0.82), and 0.80 (0.75, 0.87), respectively. For women, the HRs (95% CIs) were 0.79 (0.76, 0.82), 0.75 (0.70, 0.81), and 0.84 (0.78, 0.91), respectively. With every increase across quintiles of diet quality, there was a decrease or no change in risk of death from all-cause, CVD, and cancer for both men and women. The change in risk from all-cause and CVD mortality between quintile 1 and quintile 5 was similar for men and women. For cancer mortality, quintile 1: quintile 5 HR results supported a 20% reduction in risk of death for men compared to women, who had a 16% lower risk.

In men, when stratified by ethnicity, a protective effect was seen for quintile 1: quintile 5 for all-cause, CVD, and cancer mortality among the white, African American, and Japanese American groups, with all HR ≤ 0.83 (Supplementary Table S4). Latino men in quintile 1: quintile 5 had a reduced risk of all-cause and cancer mortality with HRs of 0.88 and 0.79, respectively, and a null association for CVD mortality. For Native Hawaiian men there was a null association for all mortality outcomes and for the tests for trend. In women, when stratified by ethnicity, a protective effect for all-cause, CVD, and cancer mortality was observed for white women in quintile 1: quintile 5 with HRs of 0.66, 0.63, and 0.71, respectively (Supplementary Table S5). African American and Japanese American women in quintile 1: quintile 5 had a reduced risk of all-cause and CVD mortality, with HR ≤ 0.72 for African American women and HR ≤ 0.87 for Japanese American women. African American and Japanese American women in quintile 1: quintile 5 had a null association for cancer mortality. Latino women in quintile 1: quintile 5 had a reduced risk of all-cause and cancer mortality, with HR ≤ 0.92, and a null association for CVD morality. There was a null association for all mortality outcomes for Native Hawaiian women and for the tests for trend. CVD mortality among Latino women, and cancer mortality among the African American and Japanese American women did not have significant tests for trend. The test for trend was statistically significant for the majority of the relationships tested.

**Figure 1:**
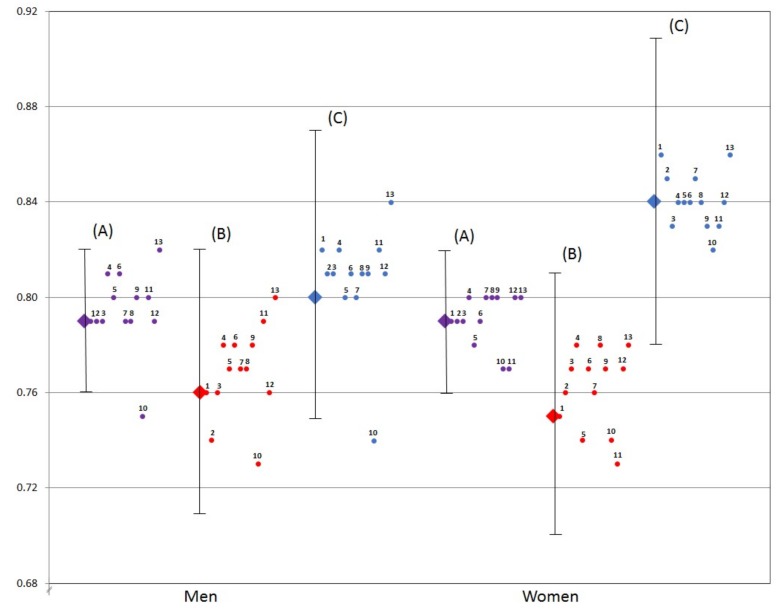
Quintile 1: quintile 5 hazard ratios (HR) and 95% confidence intervals (CI) for all-cause, cardiovascular disease (CVD) and cancer mortality for Healthy Eating Index-2015 (HEI-2015) scores with one component removed for men and women in the Multiethnic Cohort. Diamond shapes represent HR for mortality by (**A**) all-cause (purple), (**B**) CVD (red) and (**C**) cancer (blue) for men and women, as per results shown in Table 3. Smaller dots represent HR for HEI-2015 with one component removed. When excluded, the component is labeled as: (1) Total Fruits, (2) Whole Fruits, (3) Total Vegetables, (4) Greens and Beans, (5) Whole Grains, (6) Dairy, (7) Total Protein Foods, (8) Seafood and Plant Proteins, (9) Fatty Acids, (10) Refined Grains, (11) Sodium, (12) Added Sugars, (13) Saturated Fats. All models were adjusted for self-reported covariates; age at study entry, body mass index, history of diabetes, energy, ethnicity, education, marital status, smoking, weekly hours of moderate to vigorous physical activity, and alcohol intake. All models for women were also adjusted for hormone replacement therapy.

Removing any one of the 13 components from the HEI-2015 score did not substantially change the association of the index with mortality outcomes for both men and women (Figure 1 and Supplementary Tables S1–S3). Removing the Refined Grains component (component 10) changed the quintile 1: quintile 5 HR for men from 0.79 (0.76, 0.82) to 0.75 (0.72, 0.78) for all-cause mortality, 0.76 (0.71, 0.82) to 0.73 (0.68, 0.78) for CVD mortality, and 0.80 (0.75, 0.87) to 0.74 (0.68, 0.79) for cancer mortality. Repeating the same concept with the Saturated Fats component (component 13) changed the quintile 1: quintile 5 HR for men from 0.79 (0.76, 0.82) to 0.82 (0.78, 0.85) for all-cause mortality, 0.76 (0.71, 0.82) to 0.80 (0.74, 0.85) for CVD mortality, and 0.80 (0.75, 0.87) to 0.84 (0.78, 0.91) for cancer mortality. These were the largest changes seen and yet these HRs were still relatively close. When the HEI-2015 scoring standards were applied to the MEC sample with fewer mortality cases from the follow-up period of the HEI-2010 mortality analysis [19], similar associations with mortality risk were observed. The quintile 1: quintile 5 HRs were 0.78 (0.75, 0.82), 0.78 (0.72, 0.84), and 0.80 (0.73, 0.87) for all-cause, CVD, and cancer mortality, respectively, for men and 0.79 (0.75, 0.84), 0.77 (0.71, 0.85), and 0.89 (0.81, 0.97), respectively, for women. 

### 3.3. Radar Graphs

**Figure 2:**
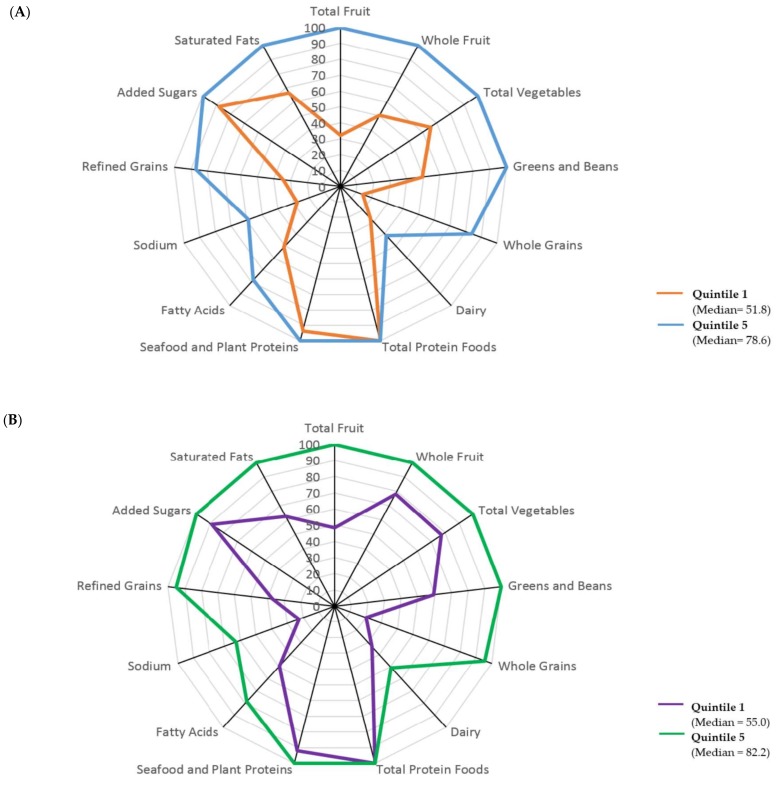
Total median scores and radar graphs of median component scores for (**A**) men and (**B**) women in Healthy Eating Index-2015 (HEI-2015) quintile 1 and quintile 5 in the Multiethnic Cohort.

Figure 2 displays radar graphs which can be used to visualize the range of intakes among the components. A perfect HEI-2015 total score (100% for each component) would be displayed as a line around the border of the radar graph. The median component scores for men in quintile 1 (the lowest quality diet group) were 50% or less for the adequacy components of Total Fruits, Whole Fruits, Greens and Beans, Whole Grains, and Dairy, as well as for the moderation components of Fatty Acids, Sodium, and Refined Grains (Figure 2A). For Total Vegetables (an adequacy component) and Saturated Fats (a moderation component), the median component scores in quintile 1 for men were between 50% and 80%. Seafood and Plant Proteins, and Total Protein Foods (adequacy components), and Added Sugars (a moderation component) were all above 80%. The lowest median component scores for men in quintile 5 were Dairy (41%), Fatty Acids (78%) and Sodium (59%). All other median component scores for men in quintile 5 were over 84%. The median component scores for women in quintile 1 were 50% or less for Total Fruits, Whole Grains, Dairy, Fatty Acids, Refined Grains, and Sodium (Figure 2B). The median component scores in quintile 1 for women were between 50% and 80% for Whole Fruits, Total Vegetables, Greens and Beans, and Saturated Fats, and over 80% for Total Protein Foods, Seafood and Plant Proteins, and Added Sugars. The lowest median component scores for women in quintile 5 were Dairy (51%), Fatty Acids (79%), and Sodium (63%). All other median component scores for women in quintile 5 were over 95%. Patterns of the median component scores were similar for men and women in quintile 1 and in quintile 5, e.g., Total Protein Foods scored the highest and Dairy scored the lowest for men and women in both quintiles. Women had higher median scores for each component, with the exception of men having higher median component scores for Seafood and Plant Proteins, Fatty Acids, Sodium, and Saturated Fats in quintile 1. Comparing the total HEI-2015 median scores between the sexes for quintile 1 and for quintile 5, women had higher median total HEI-2015 scores than men. 

## 4. Discussion

Comparing those with the lowest quality diets to those with the highest quality, the reduction in risk of mortality from all-cause, CVD, and cancer was 21%, 24%, and 20%, respectively, for men and 21%, 25%, and 16%, respectively, for women. In the HEI-2010 mortality analysis with the MEC, the reduction in the risk of mortality from all-cause, CVD, and cancer was 25%, 26%, and 24%, respectively, for men, and 21%, 23%, and 11%, respectively, for women [19]. Therefore, HRs have slightly improved for women and marginally lowered for men between the HEI-2015 and HEI-2010 mortality analyses with the MEC. When the HEI-2015 scoring standards were applied to the MEC sample with fewer mortality cases, from the follow-up period of the HEI-2010 mortality analysis, the HR results were also very similar. The reduction in risk of mortality from all-cause, CVD, and cancer was 22%, 22%, and 20%, respectively, for men and 21%, 23%, and 11%, respectively, for women. For the DPMP, standardized mortality assessments were also conducted with the HEI-2010 and the WHI-OS, and AARP cohorts. The HEI-2010 mortality analysis with the WHI-OS was conducted with 63,115 US women, of whom 83% identified as white [21,30]. The results of this study showed a reduction in risk between quintile 1: quintile 5, with HRs of 24%, 22%, and 23% for all-cause, CVD, and cancer mortality, respectively. The HEI-2010 mortality analysis for the AARP contained 242,321 men and 182,342 women from six US states and over 90% of participants identified as white [17,31]. The same mortality analysis applied to the AARP showed a reduction in risk of 22%, 15%, and 24% for all-cause, CVD, and cancer mortality, respectively, for men and 23%, 21%, and 18%, respectively, for women. The results from the HEI-2015 mortality analysis with the MEC and the HEI-2010 mortality analysis with the WHI-OS and AARP are very similar, more so than the mortality results from the HEI-2010 analyses with the MEC. For example, the reduction in risk of cancer mortality for women in the MEC, WHI-OS, and AARP were 11%, 23%, and 18%, respectively, using the HEI-2010. The reduction in risk of cancer mortality for women in the MEC changed to 16% in the HEI-2015 analysis. A recent meta-analysis on the association between the HEI-2005/HEI 2010, AHEI, and DASH and health outcomes for both men and women had similar findings to this HEI-2015 analysis with the MEC [32]. Consistency between the HEI-2010 analyses and this HEI-2015 analysis reinforces the use of HEI as an assessment of diet quality.

The analysis that removed one component at a time did not change the protective association of the remaining HEI components. Therefore, no one HEI component made an independent significant contribution to the total score (Figure 1). The Saturated Fat component did distinguish itself, as its removal changed the HRs slightly towards the null for all three mortality outcomes among men only. Previous versions of the HEI included Saturated Fat as part of the empty calories component comprised of solid fat, added sugars, and alcohol [3]. The HEI-2015 offers the first opportunity to evaluate the Added Sugars and Saturated Fat components independently, however alcohol is now part of total energy intake. Removal of the Added Sugars component had no influence on moving the hazard ratios. On the other hand, the component whose removal consistently changed the HRs away from the null was Refined Grains for all-cause mortality, CVD mortality, and cancer mortality among men only, although this shift was minor and did not change the overall results. The by-component models using HEI-2010 among the members of the AARP cohort reported the unexpected finding of an increase in risk for all-cause mortality among men and women with higher scores for the Refined Grain component (indicating lower consumption) [16]. Future analysis might consider reconciling these observations across the HEI-2010 and HEI-2015 and the cohorts involved in the DPMP. These results support and reinforce the multidimensionality of the HEI and the representation of diet quality using a wide array of components.

Higher diet quality was associated with improved mortality outcomes in this analysis among a multiethnic population of men and women. All of the HEI-2015 components contributed to the association of diet quality and mortality. Given this, dietary components needing improvement for people with the lowest quality diets could be emphasized in public health messages. The components with the largest differences in median scores between quintile 1 and quintile 5 were identified as Total Fruits, Whole Fruits, Greens and Beans, Whole Grains, and Refined Grains for men and Total Fruits, Whole Grains, and Refined Grains for women (Figure 2). The results of the present analysis suggest increasing the intake of foods that fall into these components may improve mortality outcomes for people with the lowest quality diets. Data from the 2007–2010 National Health and Nutrition Examination Survey (NHANES) reported that men and women 31 years and older in the US do not meet the requirements for fruits, vegetables, and whole grains and exceed the recommended intake of refined grains [4,33]. The message on increasing the intake of fruits, vegetables, and whole grains to improve health outcomes is consistent with results from both the MEC and NHANES [4,33]. 

The components with the lowest median scores were the same for people in quintile 1 and quintile 5. These components were Dairy, Fatty Acids, and Sodium, with median scores of less than 50%. Improving scores for Dairy, Fatty Acids, and Sodium components may help to improve mortality outcomes for people with the lowest quality diets. The current mortality analysis does not provide evidence on whether increasing Dairy, Fatty Acids, and Sodium component scores for people with the highest quality diets will offer any additional protection. The DGA reports, on average, that adults in the US are not achieving their recommended intakes of dairy and oils, and average intakes of saturated fats and sodium are met or exceeded [4]. The MEC results and the DGAs support evidence that people with low quality diets should increase their intakes of these foods to improve health outcomes [4,33]. The current dietary guidelines also promote replacing SFAs with PUFAs to reduce CVD-related deaths and decreasing sodium intake to reduce CVD events [4]. Based on this evidence from the DGAs and the MEC, people with the highest quality diets may have improved mortality outcomes if they meet recommended intakes of SFAs, PUFAs, and sodium. 

The median component scores for men and women in the MEC in quintile 1 and quintile 5 were at 100% (a perfect score) for the Total Protein Foods component. Similarly, results from NHANES 2007–2010 support that mean intakes of meat, poultry, and eggs for adults 31 years and older are at recommended levels for women and at or above recommended levels for men [4,33]. The standard for a perfect score for Total Protein Foods is ≥2.5 oz per 1000 kcal per day. There is no upper limit for Total Protein Foods using this scoring standard; therefore, we do not know if consuming more than 2.5 oz of Total Protein Foods per 1000 kcal per day further improves or worsens mortality outcomes. Also, we do not know if consuming Total Protein Foods in excess replaces intake of foods found in the other 12 components, which would lower these component scores. Having no upper limit for component scoring standards may be a limitation of the HEI. 

The median component scores for quintile 1 and quintile 5 for Added Sugars for men and women in the MEC were each above 88%. In comparison, the average intake of added sugar for adults in NHANES were all above the recommended maximum limit [4,33]. Previous research on the MEC found that Japanese Americans had the greatest percentage of people who met the DGA recommendations for added sugars [34,35]. The MEC has a large proportion of Japanese Americans (26.4%), which may explain why median component scores for Added Sugars indicate a low intake in the MEC compared to excessive average intakes in NHANES. For the Seafood and Plant Proteins component, the median scores among the MEC men and women in both the 1st and 5th quintiles were above 80%. Results reported from NHANES 2007-2010 indicated intakes of nuts, seeds, and soy products were at or above recommended intakes among adults, whereas seafood intakes were below recommendations [4,33]. Previous research shows that Native Hawaiians and Japanese Americans in the MEC have more servings of fish per day than other ethnic groups [34,35]. These two ethnic groups make up 1/3 of the MEC sample; therefore, their seafood intake may contribute to the median component scores of over 80% for Seafood and Plant Proteins. 

The overall mean HEI-2015 score among men and women in the MEC, as estimated using the FFQ, was 65 and 69, respectively. Among men, Native Hawaiian men had the lowest score at 63 compared to African American and white men, with scores of 67. For women, the range was 71 among African American and white women to 67 among Native Hawaiian women. At the time of this paper, no published information about HEI-2015 scores among adults or children had been published. For any comparison, HEI-2015 scores derived from a FFQ would be preferable, as was done by Liese et al. [15]. Although the mean scores by ethnic group were almost all statistically significantly different, the range of scores was small, i.e., four points for both men and women. An examination of component scores between ethnic groups to further explore variation in dietary exposures is warranted.

Identifying the characteristics of people with the lowest quality diets may help to further tailor nutrition education messages. Comparing the results across the WHI-OS, AARP, and MEC cohorts, college graduates were more likely to be classified as having a higher quality diet based on HEI-2010 [16,21]. In the AARP and MEC cohorts, women had slightly higher diet quality scores than men [16]. For both men and women in the MEC, a higher percentage of Japanese Americans, Native Hawaiians, and Latinos were in quintile 1 and a higher percentage of whites and African Americans were in quintile 5. The WHI-OS cohort is comprised of women and similar to the women in the MEC cohort, there was a higher percentage of whites in quintile 5 compared to quintile 1 and a higher percentage of Hispanic women in quintile 1 compared to quintile 5 [21]. In the WHI-OS, a greater percentage of Black women were in quintile 1 compared to quintile 5, which is in contrast to the MEC, where a larger proportion of Black women were in quintile 5 [21]. Thus, the quality of diet within any one group may vary by geographic area. 

Of note, the highest versus the lowest HEI-2015 scores were consistently more protective for white and African Americans. The development of the HEI is guided by evidence-based research, and nutrition research is dominated by studies conducted among white and African American participants [36,37,38,39]. This may account for why the HEI-2015 performs better for white and African Americans. In this analysis, Native Hawaiian men and women had a null association between HEI-2015 quintile 1: quintile 5 and all-cause, CVD, and cancer mortality. Native Hawaiians have the lowest sample size compared to all other ethnic groups in the MEC. Therefore, the power for the analyses of diet quality and mortality outcomes in Native Hawaiians may not be large enough to draw significant findings. Latino men and women also had null associations between HEI-2015 quintile 1: quintile 5 and CVD mortality. Latinos in the US have the lowest rate of CVD compared to other ethnic groups [40]. These rates are mimicked in this current analysis, with Latino men and women making up 25% and 22% of this MEC sample, respectively, but having the second lowest rate of CVD mortality behind Native Hawaiians. Therefore, the relatively lower rate of CVD mortality in Latino men and women may be contributing to the null association between diet quality and CVD mortality. The associations between the HEI-2015 and mortality outcomes by ethnic group in the MEC are similar to those found in the HEI-2010 mortality analysis with the MEC [19].

A limitation of this study was the use of a food frequency questionnaire (FFQ) to collect dietary information at baseline, which could introduce bias [22]. However, this QFFQ was validated and calibrated in each ethnic-sex group and the correlations between the QFFQ and 24-hour dietary recalls were 0.55–0.74 for energy adjusted nutrients [22]. Another limitation was dietary data only being assessed once at baseline; therefore, this analysis was not able to capture the influence of dietary changes on mortality outcomes. In addition, all demographic and anthropometric variables were self-reported, and we cannot rule out whether other factors not measured and controlled for, could have affected mortality outcomes; e.g., access to health care. Participants in the MEC were recruited from Hawaii and LA; therefore, results of this study may not be generalizable outside of these areas. Lastly, measurement error is an important consideration relevant to all self-reported behavioral variables. The simple models used to examine predictive validity do not address measurement error; however, efforts are underway to do so for future analyses.

The strengths of this study include the use of a large multiethnic sample that was followed prospectively for over 17 years and the use of a comprehensive QFFQ that was designed to capture ethnic specific foods, allowing for this multiethnic comparison. In addition, covariate data at baseline were collected for almost every participant, permitting the adjustment for multiple, salient risk factors. This study also applied the same standardized regression analysis previously used in diet quality and mortality analysis by the DPMP. Using this standardized approach allows comparisons to be made between the present study and previous and future DPMP analyses of dietary indices and mortality outcomes. For example, once finalized, comparisons can be made between the MEC, the WHI-OS and the AARP mortality analysis with the HEI-2015.

## 5. Conclusions

In summary, people in the MEC with higher HEI-2015 scores had a reduction in risk of all-cause, CVD, and cancer mortality. Updates to component scoring between the HEI-2010 and HEI-2015 do not appear to have affected the balance of the scoring system, and mortality analysis results were robust between studies that used either index. Increasing intake of whole fruits, vegetables, whole grains, MUFA, PUFA and reducing intake of refined grains, sodium, and saturated fats may improve dietary patterns and mortality outcomes for people with the poorest quality diets. Improving intake as a whole versus any single food group appears to be most beneficial in reducing risk for mortality. The HEI-2015 is a useful means by which to measure diet quality. 

## Figures and Tables

**Table 1:** Healthy Eating Index-2015 (HEI-2015) component scoring standards using standardized cup and ounce equivalents from the MPED **^1,2,3^**.

Components	Maximum Scores	Standard for Maximum Scores ^3^	Standard for Minimum of Zero
Adequacy			
Total Fruits ^4^	5	≥0.8 cup	No Fruits
Whole Fruits ^5^	5	≥0.4 cup	No Whole Fruits
Total Vegetables ^6^	5	≥1.1 cup	No Vegetables
Greens & Beans ^6^	5	≥0.2 cup	No Greens and Beans
Whole Grains	10	≥1.5 oz	No Whole Grains
Dairy ^7^	10	≥1.3 cup	No Dairy
Total Protein Foods ^6,8^	5	≥2.5 oz	No Protein Foods
Seafood & Plant Proteins ^6,8^	5	≥0.8 cup	No Seafood & Plant Proteins
Fatty Acids ^9^	10	(PUFAs + MUFAs)/SFAs ≥ 2.5	(PUFAs + MUFAs)/SFAs ≤ 1.2
Moderation			
Refined Grains	10	≤1.8 oz	≥4.3 oz
Sodium	10	≤1.1 g	≥2.0 g
Added Sugars	10	≤6.5% of energy	≥26% of energy
Saturated Fats	10	≤8% of energy	≥16% of energy

^1^ Scoring standards are expressed as cup and ounce equivalents from the MyPyramid Equivalents Database (MPED), whereby 1 oz = 28.3 g and 1 cup = 225 mL. ^2^ Intakes between the minimum and maximum standards are scored proportionately. ^3^ All standards represent amounts per 1000 kcal, except for Fatty Acids, Added Sugars and Saturated Fats. ^4^ Includes 100% fruit juice. ^5^ Includes all forms expect juice. ^6^ Includes legumes (beans and peas). ^7^ Includes all milk products, such as fluid milk, yogurt, cheese and fortified soy beverages. ^8^ Includes seafood, nuts, seeds, and soy products (other than beverages). ^9^ Ratio of poly- and mono-unsaturated fatty acids (PUFAs and MUFAs) to saturated fatty acids (SFAs).

**Table 2:** Descriptive characteristics of participants (*n* = 156,804) in the Multiethnic Cohort by quintiles of Healthy Eating Index-2015 (HEI-2015) scores.

	Quintile 1	Quintile 2	Quintile 3	Quintile 4	Quintile 5
**Men (*n* = 70,170)**					
HEI-2015 scores, range	17.9–56.1	56.2–62.1	62.2–67.5	67.6–74.0	74.1–98.7
Mean HEI-2015 score ***	50.7	59.4	64.9	70.7	79.6
*N*	14,034	14,034	14,035	14,033	14,034
Mortality, *n* cases	5003	5177	5321	5355	5520
Cardiovascular disease	1708	1740	1864	1894	1924
Cancer	1566	1598	1572	1561	1515
Age at time of death, years ^1^	74.4 ± 9.6	75.9 ± 9.4	77.0 ± 9.2	77.9 ± 9.1	79.3 ± 8.6
Age at time of questionnaire, years ^1,^***	56.8 ± 8.6	58.3 ± 8.7	59.4 ± 8.7	60.0 ± 8.7	61.1 ± 8.6
Ethnicity, % of row					
Japanese American (*n* = 21,239)	24.1	21.5	20.0	17.8	16.6
Latino (*n* = 17,595)	20.7	23.1	22.8	19.8	13.7
White (*n* = 17,330)	14.9	16.3	18.0	23.0	28.0
African American (*n* = 9014)	15.1	17.0	19.2	21.7	26.9
Native Hawaiian (*n* = 4992)	27.0	20.9	18.7	17.2	16.3
Body mass index (kg/m^2^) ^1,^***	26.9 ± 4.5	26.9 ± 4.3	26.8 ± 4.2	26.6 ± 4.1	26.1 ± 3.8
Energy intake, kcal ^1,^***	2479 ± 1158	2552 ± 1220	2524 ± 1181	2448 ± 1107	2256 ± 978
Physical activity, h/week ^1,2,^***	1.2 ± 1.5	1.3 ± 1.6	1.4 ± 1.5	1.4 ± 1.5	1.5 ± 1.5
History of diabetes, % with diabetes ***	8.4	10.0	11.4	11.7	12.5
Smoking, % who never smoked ***	24.3	28.0	30.6	33.8	37.7
Education, % graduated from college ***	23.9	26.2	28.6	33.3	38.5
Marital status, % married	74.5	76.3	77.7	76.8	75.2
**Women (*n* = 86,634)**					
HEI-2015 scores, range	23.5–59.8	59.9–66.3	66.4–71.8	71.9–78.0	78.1–99.8
Mean HEI-2015 score ***	53.8	63.3	69.2	74.9	83.0
*N*	17,327	17,326	17,328	17,327	17,326
Mortality, *n* cases	4603	4809	5020	5119	5515
Cardiovascular disease	1493	1637	1747	1782	1873
Cancer	1398	1365	1385	1353	1465
Age at time of death, years	75.1 ± 9.9	77.3 ± 9.6	78.3 ± 9.1	79.5 ± 9.1	80.4 ± 8.6
Age at time of questionnaire, years ***	56.4 ± 8.6	58.2 ± 8.7	59.3 ± 8.7	60.1 ± 8.8	61.5 ± 8.5
Ethnicity, % of row					
Japanese American (*n* = 24,785)	21.1	21.5	20.1	19.0	18.3
White (*n* = 20,653)	15.8	16.6	19.7	22.9	25.0
Latina (*n* = 18,756)	25.4	24.1	21.2	17.0	12.4
African American (*n* = 16,072)	15.0	17.0	19.3	22.3	26.5
Native Hawaiian (*n* = 6368)	26.1	20.8	18.8	17.8	16.5
Body mass index (kg/m^2^) ***	27.1 ± 6.2	26.7 ± 5.8	26.5 ± 5.6	26.1 ± 5.4	25.5 ± 5.2
Energy intake, kcal ***	2052 ± 1068	2038 ± 1023	2003 ± 967	1956 ± 915	1865 ± 817
Physical activity, h/week ^2,^***	1.0 ± 1.2	1.0 ± 1.3	1.1 ± 1.3	1.2 ± 1.3	1.3 ± 1.3
History of diabetes, % with diabetes ***	8.2	9.3	9.8	9.5	10.1
Smoking, % never smoked ***	50.5	55.8	56.5	57.9	58.7
Education, % graduated from college ***	18.7	21.3	23.9	27.4	31.3
Marital status, % married *	59.1	60.9	60.5	59.4	57.6
Hormone replacement therapy, % users ***	37.4	42.7	46.1	49.2	53.1

*** *p* value < 0.001 for independent sample *t*-test between quintile 1 and quintile 5 for quantitative variables and test of proportions for discrete variables collected at baseline. ^1^ Mean ± SD (all such values). * *p* value < 0.05 between quintile 1 and quintile 5, for test of proportions for discrete variables collected at baseline. ^2^ Represents self-reported weekly hours of moderate to vigorous physical activity.

**Table 3:** Hazard ratios (HR) (95% confidence intervals (CI)) for all-cause, cardiovascular disease (CVD), and cancer mortality according to quintiles of Healthy Eating Index-2015 (HEI-2015) scores in men (*n* = 70,170) and women (*n* = 86,634) in the Multiethnic Cohort ^1^.

HEI-2015 Category	*n*	Any Deaths *n*	Person-Years of Follow-Up	All-Cause Mortality ^1^ HR (95% CI)	CVD Deaths *n*	CVD Mortality ^1^ HR (95% CI)	Cancer Deaths *n*	Cancer Mortality ^1^ HR (95% CI)
**Men ^2,3^**								
Quintile 1	14,034	5003	252,098	1.00	1708	1.00	1566	1.00
Quintile 2	14,034	5177	250,512	0.93 (0.90, 0.97)	1740	0.90 (0.84, 0.96)	1598	0.97 (0.90, 1.04)
Quintile 3	14,035	5321	250,072	0.89 (0.85, 0.92)	1864	0.88 (0.82, 0.94)	1572	0.91 (0.85, 0.98)
Quintile 4	14,033	5355	250,974	0.85 (0.81, 0.88)	1894	0.84 (0.78, 0.90)	1561	0.88 (0.82, 0.95)
Quintile 5	14,034	5520	252,489	0.79 (0.76, 0.82)	1924	0.76 (0.71, 0.82)	1515	0.80 (0.75, 0.87)
**Women ^3,4^**								
Quintile 1	17,327	4603	327,551	1.00	1493	1.00	1398	1.00
Quintile 2	17,326	4809	327,362	0.92 (0.89, 0.96)	1637	0.93 (0.87, 1.00)	1365	0.93 (0.86, 1.00)
Quintile 3	17,328	5020	325,938	0.87 (0.84, 0.91)	1747	0.88 (0.82, 0.95)	1385	0.90 (0.83, 0.97)
Quintile 4	17,327	5119	326,802	0.82 (0.79, 0.86)	1782	0.83 (0.77, 0.89)	1353	0.84 (0.78, 0.91)
Quintile 5	17,326	5515	325,836	0.79 (0.76, 0.82)	1873	0.75 (0.70, 0.81)	1465	0.84 (0.78, 0.91)

^1^ *p*-trend < 0.0001 for all models. ^2^ Adjusted for self-reported covariates; age at study entry, body mass index, history of diabetes, energy, ethnicity, education, marital status, smoking, weekly hours of moderate to vigorous physical activity, and alcohol intake. ^3^ Quintile 1 is lowest score and quintile 5 is highest score, HEI ranges shown in Table 2. ^4^ Adjusted for self-reported covariates; age at study entry, body mass index, history of diabetes, energy, ethnicity, education, marital status, smoking, physical activity, hormone replacement therapy, and alcohol intake.

## Supplementary Materials

The following are available online at http://www.mdpi.com/2072-6643/10/4/452/s1. Table S1: Hazard ratios (HR) (95% confidence intervals (CI)) for all-cause mortality according to quintiles of Healthy Eating Index-2015 (HEI-2015) scores with one component removed for men and women in the Multiethnic Cohort, Table S2: Hazard ratios (HR) (95% confidence intervals (CI)) for cardiovascular disease mortality according to quintiles of Healthy Eating Index-2015 (HEI-2015) scores with one component removed for men and women in the Multiethnic Cohort, Table S3: Hazard ratios (HR) (95% confidence intervals (CI)) for cancer mortality according to quintiles of Healthy Eating Index-2015 (HEI-2015) with one component removed for men and women in the Multiethnic Cohort, Table S4: Hazard ratios (HR) (95% confidence intervals (CI)) for all-cause, cardiovascular disease (CVD), and cancer mortality according to quintiles of Healthy Eating Index-2015 (HEI-2015) scores in men (*n* = 70,170) stratified by ethnicity in the Multiethnic Cohort, Table S5: Hazard ratios (HR) (95% confidence intervals (CI)) for all-cause, cardiovascular disease (CVD), and cancer mortality according to quintiles of Healthy Eating Index-2015 (HEI-2015) scores in women (*n* = 86,634) stratified by ethnicity in the Multiethnic Cohort.

## Appendix A

**Table A1:** Estimated mean and standard deviation of HEI-2015 total scores by ethnic group and sex, the Multiethnic Cohort ^a^.

Ethnic Group	Men	*n*	Women ****	*n*
White	67.4 ± 10.4 *	17,330	70.5 ± 10.4	20,653
African American	67.1 ± 10.4 *	9014	70.9 ± 10.3	16,072
Native Hawaiian	63.2 ± 10.6 **	4992	67.1 ± 10.9	6368
Japanese American	63.7 ± 10.3 ***	21,239	68.3 ± 10.3	24,785
Latino	63.8 ± 9.3 ***	17,595	66.5 ± 9.8	18,756
Total group	65.0 ± 10.3	70,170	68.8 ± 10.4	86,634

^a^ Results based on responses to food frequency questionnaire. * *p* < 0.01 different from Native Hawaiian, Japanese American, Latino; ** *p* < 0.01 different from all others; *** *p* < 0.01 different from Native Hawaiian, African American, white. **** Among women, each ethnic group significantly different from all others at *p* < 0.01.
