# Supplementary material for: Testing the Predictive Validity of the Healthy Eating Index-2015 in the Multiethnic Cohort: Is the Score Associated with a Reduced Risk of All-Cause and Cause-Specific Mortality?
Source: Nutrients. 2018 Apr 5;10(4):452. doi: 10.3390/nu10040452 (PMC5946237; doi:10.3390/nu10040452)
Supplement: Supplementary file 1 [file nutrients-10-00452-s001.pdf]

nutrients

MDPI

Supplementary Materials

Table S1. Hazard ratios (HR) (95% confidence intervals (CI)) for all-cause mortality according to quintiles of Healthy Eating Index-2015 (HEI-2015) scores with one component removed for men and women in the Multiethnic Cohort [1].

Component removed from HEI-2015 score	Quintile 1 HR	Quintile 2 HR (95% CI)	Quintile 3 HR (95% CI)	Quintile 4 HR (95% CI)	Quintile 5 HR (95% CI)
Men [2]					
Total Fruits	1.000	0.949 (0.912, 0.986)	0.886 (0.852, 0.922)	0.845 (0.813, 0.880)	0.793 (0.761, 0.825)
Whole Fruits	1.000	0.928 (0.892, 0.965)	0.881 (0.847, 0.916)	0.842 (0.809, 0.876)	0.787 (0.756, 0.819)
Total Vegetables	1.000	0.942 (0.905, 0.980)	0.902 (0.867, 0.938)	0.850 (0.816, 0.884)	0.795 (0.763, 0.828)
Greens & Beans	1.000	0.942 (0.906, 0.980)	0.898 (0.863, 0.934)	0.854 (0.820, 0.889)	0.806 (0.774, 0.840)
Whole Grains	1.000	0.941 (0.905, 0.979)	0.872 (0.838, 0.907)	0.843 (0.810, 0.877)	0.798 (0.767, 0.830)
Dairy	1.000	0.944 (0.908, 0.982)	0.910 (0.875, 0.947)	0.844 (0.811, 0.878)	0.808 (0.776, 0.841)
Total Protein Foods	1.000	0.938 (0.902, 0.976)	0.898 (0.863, 0.934)	0.839 (0.806, 0.874)	0.793 (0.761, 0.825)
Seafood & Plant Proteins	1.000	0.944 (0.908, 0.982)	0.891 (0.857, 0.928)	0.845 (0.812, 0.879)	0.793 (0.761, 0.826)
Fatty Acids	1.000	0.938 (0.902, 0.976)	0.893 (0.858, 0.929)	0.857 (0.823, 0.893)	0.805 (0.772, 0.839)
Refined Grains	1.000	0.903 (0.869, 0.939)	0.864 (0.831, 0.898)	0.816 (0.785, 0.849)	0.748 (0.719, 0.779)
Sodium	1.000	0.923 (0.887, 0.960)	0.878 (0.844, 0.913)	0.849 (0.816, 0.883)	0.801 (0.769, 0.834)
Added Sugars	1.000	0.934 (0.898, 0.972)	0.892 (0.858, 0.928)	0.843 (0.810, 0.878)	0.793 (0.761, 0.826)
Saturated Fats	1.000	0.936 (0.899, 0.974)	0.908 (0.873, 0.945)	0.882 (0.847, 0.918)	0.816 (0.783, 0.851)
Women [3]					
Total Fruits	1.000	0.917 (0.880, 0.955)	0.882 (0.847, 0.919)	0.821 (0.788, 0.853)	0.789 (0.757, 0.823)
Whole Fruits	1.000	0.907 (0.870, 0.945)	0.882 (0.847, 0.919)	0.819 (0.786, 0.853)	0.789 (0.757, 0.823)
Total Vegetables	1.000	0.917 (0.880, 0.956)	0.875 (0.840, 0.912)	0.836 (0.802, 0.871)	0.792 (0.759, 0.825)
Greens & Beans	1.000	0.939 (0.901, 0.978)	0.882 (0.846, 0.919)	0.847 (0.812, 0.883)	0.804 (0.771, 0.838)
Whole Grains	1.000	0.897 (0.861, 0.935)	0.864 (0.829, 0.900)	0.834 (0.801, 0.869)	0.778 (0.747, 0.811)
Dairy	1.000	0.926 (0.889, 0.965)	0.887 (0.851, 0.924)	0.841 (0.807, 0.877)	0.792 (0.760, 0.826)
Total Protein Foods	1.000	0.930 (0.892, 0.969)	0.879 (0.844, 0.917)	0.838 (0.804, 0.874)	0.798 (0.765, 0.832)
Seafood & Plant Proteins	1.000	0.930 (0.892, 0.969)	0.879 (0.844, 0.916)	0.834 (0.800, 0.869)	0.799 (0.767, 0.834)
Fatty Acids	1.000	0.935 (0.897, 0.974)	0.887 (0.851, 0.924)	0.837 (0.803, 0.873)	0.802 (0.769, 0.837)
Refined Grains	1.000	0.910 (0.874, 0.947)	0.864 (0.830, 0.900)	0.805 (0.772, 0.838)	0.771 (0.740, 0.804)
Sodium	1.000	0.902 (0.866, 0.940)	0.844 (0.810, 0.879)	0.825 (0.792, 0.860)	0.774 (0.743, 0.807)
Added Sugars	1.000	0.950 (0.912, 0.990)	0.888 (0.853, 0.926)	0.843 (0.808, 0.879)	0.804 (0.771, 0.839)
Saturated Fats	1.000	0.934 (0.896, 0.973)	0.883 (0.847, 0.920)	0.834 (0.800, 0.870)	0.804 (0.771, 0.839)

[1] *P*-trend < 0.0001 for all models. [2] Adjusted for self-reported covariates; age at study entry, body mass index, history of diabetes, energy, ethnicity, education, marital status, smoking, weekly hours of moderate to vigorous physical activity, and alcohol intake. [3] Adjusted for self-reported covariates; age at study entry, body mass index, history of diabetes, energy, ethnicity, education, marital status, smoking, weekly hours of moderate to vigorous physical activity, hormone replacement therapy, and alcohol intake.

Table S2. Hazard ratios (HR) (95% confidence intervals (CI)) for cardiovascular disease mortality according to quintiles of Healthy Eating Index-2015 (HEI-2015) scores with one component removed for men and women in the Multiethnic Cohort [1].

Component removed from HEI-2015 score	Quintile 1 HR	Quintile 2 HR (95% CI)	Quintile 3 HR (95% CI)	Quintile 4 HR (95% CI)	Quintile 5 HR (95% CI)
Men [2]					
Total Fruits	1.000	0.916 (0.857, 0.980)	0.858 (0.803, 0.917)	0.830 (0.776, 0.888)	0.759 (0.709, 0.813)
Whole Fruits	1.000	0.878 (0.821, 0.939)	0.840 (0.785, 0.898)	0.829 (0.776, 0.886)	0.742 (0.693, 0.795)
Total Vegetables	1.000	0.903 (0.844, 0.966)	0.881 (0.823, 0.942)	0.845 (0.790, 0.904)	0.756 (0.706, 0.810)
Greens & Beans	1.000	0.907 (0.848, 0.970)	0.883 (0.826, 0.945)	0.852 (0.796, 0.912)	0.775 (0.723, 0.830)
Whole Grains	1.000	0.927 (0.867, 0.991)	0.870 (0.814, 0.930)	0.833 (0.779, 0.891)	0.769 (0.718, 0.823)
Dairy	1.000	0.937 (0.876, 1.002)	0.892 (0.835, 0.954)	0.837 (0.782, 0.895)	0.779 (0.727, 0.834)
Total Protein Foods	1.000	0.918 (0.857, 0.982)	0.890 (0.832, 0.952)	0.841 (0.786, 0.900)	0.766 (0.715, 0.821)
Seafood & Plant Proteins	1.000	0.925 (0.864, 0.990)	0.888 (0.830, 0.950)	0.855 (0.799, 0.915)	0.772 (0.720, 0.828)
Fatty Acids	1.000	0.925 (0.864, 0.990)	0.877 (0.819, 0.939)	0.863 (0.806, 0.924)	0.785 (0.732, 0.842)
Refined Grains	1.000	0.902 (0.844, 0.964)	0.848 (0.793, 0.907)	0.834 (0.780, 0.891)	0.730 (0.682, 0.782)
Sodium	1.000	0.899 (0.840, 0.962)	0.864 (0.807, 0.924)	0.867 (0.811, 0.927)	0.791 (0.739, 0.848)
Added Sugars	1.000	0.900 (0.841, 0.963)	0.864 (0.808, 0.924)	0.843 (0.788, 0.902)	0.758 (0.708, 0.812)
Saturated Fats	1.000	0.927 (0.866, 0.993)	0.906 (0.846, 0.970)	0.884 (0.825, 0.947)	0.796 (0.742, 0.854)
Women [3]					
Total Fruits	1.000	0.921 (0.857, 0.988)	0.886 (0.825, 0.950)	0.818 (0.762, 0.879)	0.750 (0.698, 0.806)
Whole Fruits	1.000	0.923 (0.860, 0.992)	0.904 (0.842, 0.970)	0.820 (0.764, 0.881)	0.754 (0.702, 0.811)
Total Vegetables	1.000	0.947 (0.881, 1.017)	0.900 (0.838, 0.966)	0.831 (0.773, 0.893)	0.771 (0.717, 0.829)
Greens & Beans	1.000	0.965 (0.899, 1.037)	0.899 (0.836, 0.965)	0.839 (0.780, 0.902)	0.779 (0.725, 0.838)
Whole Grains	1.000	0.896 (0.834, 0.962)	0.874 (0.814, 0.938)	0.839 (0.782, 0.901)	0.734 (0.683, 0.788)
Dairy	1.000	0.946 (0.880, 1.017)	0.905 (0.843, 0.972)	0.850 (0.791, 0.913)	0.770 (0.716, 0.828)
Total Protein Foods	1.000	0.943 (0.878, 1.013)	0.876 (0.816, 0.941)	0.839 (0.781, 0.902)	0.763 (0.709, 0.820)
Seafood & Plant Proteins	1.000	0.977 (0.910, 1.050)	0.898 (0.836, 0.965)	0.838 (0.780, 0.901)	0.775 (0.720, 0.834)
Fatty Acids	1.000	0.957 (0.890, 1.028)	0.901 (0.839, 0.968)	0.845 (0.786, 0.908)	0.772 (0.718, 0.831)
Refined Grains	1.000	0.902 (0.841, 0.967)	0.894 (0.834, 0.958)	0.777 (0.724, 0.834)	0.737 (0.686, 0.791)
Sodium	1.000	0.919 (0.856, 0.987)	0.871 (0.812, 0.935)	0.840 (0.783, 0.902)	0.728 (0.678, 0.782)
Added Sugars	1.000	0.976 (0.909, 1.048)	0.899 (0.837, 0.966)	0.840 (0.781, 0.903)	0.770 (0.716, 0.828)
Saturated Fats	1.000	0.956 (0.890, 1.028)	0.902 (0.840, 0.970)	0.830 (0.772, 0.893)	0.778 (0.723, 0.837)

[1] *P*-trend < 0.0001 for all models. [2] Adjusted for self-reported covariates; age at study entry, body mass index, history of diabetes, energy, ethnicity, education, marital status, smoking, weekly hours of moderate to vigorous physical activity, and alcohol intake. [3] Adjusted for self-reported covariates; age at study entry, body mass index, history of diabetes, energy, ethnicity, education, marital status, smoking, weekly hours of moderate to vigorous physical activity, hormone replacement therapy, and alcohol intake.

Table S3. Hazard ratios (HR) (95% confidence intervals (CI)) for cancer mortality according to quintiles of Healthy Eating Index-2015 (HEI-2015) with one component removed for men and women in the Multiethnic Cohort [1].

**Component removed from HEI-2015 score**	**Quintile 1 HR**	**Quintile 2 HR (95% CI)**	**Quintile 3 HR (95% CI)**	**Quintile 4 HR (95% CI)**	**Quintile 5 HR (95% CI)**
Men [2]					
Total Fruits	1.000	0.986 (0.919, 1.058)	0.930 (0.866, 0.999)	0.887 (0.825, 0.953)	0.815 (0.757, 0.878)
Whole Fruits	1.000	0.963 (0.897, 1.034)	0.944 (0.879, 1.013)	0.873 (0.812, 0.938)	0.811 (0.752, 0.873)
Total Vegetables	1.000	0.953 (0.888, 1.024)	0.929 (0.865, 0.998)	0.868 (0.808, 0.934)	0.811 (0.752, 0.874)
Greens & Beans	1.000	0.954 (0.888, 1.024)	0.928 (0.864, 0.997)	0.876 (0.815, 0.942)	0.816 (0.757, 0.880)
Whole Grains	1.000	0.930 (0.867, 0.998)	0.895 (0.834, 0.961)	0.863 (0.803, 0.927)	0.804 (0.747, 0.865)
Dairy	1.000	0.901 (0.839, 0.968)	0.922 (0.859, 0.989)	0.858 (0.799, 0.922)	0.807 (0.750, 0.869)
Total Protein Foods	1.000	0.962 (0.896, 1.032)	0.925 (0.861, 0.993)	0.863 (0.803, 0.929)	0.800 (0.742, 0.862)
Seafood & Plant Proteins	1.000	0.951 (0.886, 1.021)	0.923 (0.860, 0.992)	0.849 (0.789, 0.913)	0.807 (0.749, 0.870)
Fatty Acids	1.000	0.955 (0.890, 1.026)	0.931 (0.867, 1.000)	0.847 (0.787, 0.912)	0.812 (0.753, 0.876)
Refined Grains	1.000	0.869 (0.811, 0.932)	0.875 (0.816, 0.938)	0.828 (0.771, 0.889)	0.736 (0.684, 0.793)
Sodium	1.000	0.916 (0.853, 0.983)	0.922 (0.859, 0.989)	0.848 (0.789, 0.912)	0.825 (0.766, 0.888)
Added Sugars	1.000	0.975 (0.909, 1.046)	0.917 (0.854, 0.985)	0.858 (0.798, 0.923)	0.811 (0.753, 0.874)
Saturated Fats	1.000	0.971 (0.904, 1.043)	0.945 (0.879, 1.016)	0.907 (0.843, 0.976)	0.841 (0.780, 0.907)
Women [3]					
Total Fruits	1.000	0.978 (0.907, 1.055)	0.919 (0.852, 0.992)	0.849 (0.785, 0.918)	0.861 (0.796, 0.932)
Whole Fruits	1.000	0.946 (0.877, 1.020)	0.903 (0.837, 0.975)	0.841 (0.778, 0.910)	0.849 (0.785, 0.919)
Total Vegetables	1.000	0.922 (0.854, 0.995)	0.897 (0.832, 0.969)	0.837 (0.774, 0.905)	0.832 (0.769, 0.900)
Greens & Beans	1.000	0.932 (0.864, 1.006)	0.901 (0.835, 0.973)	0.862 (0.797, 0.931)	0.838 (0.774, 0.906)
Whole Grains	1.000	0.925 (0.858, 0.998)	0.901 (0.835, 0.972)	0.842 (0.779, 0.909)	0.837 (0.774, 0.904)
Dairy	1.000	0.933 (0.865, 1.007)	0.909 (0.842, 0.981)	0.849 (0.785, 0.918)	0.841 (0.778, 0.909)
Total Protein Foods	1.000	0.941 (0.872, 1.016)	0.916 (0.849, 0.988)	0.840 (0.777, 0.908)	0.849 (0.785, 0.918)
Seafood & Plant Proteins	1.000	0.928 (0.860, 1.002)	0.904 (0.837, 0.975)	0.839 (0.776, 0.907)	0.840 (0.777, 0.909)
Fatty Acids	1.000	0.953 (0.884, 1.028)	0.898 (0.832, 0.970)	0.824 (0.762, 0.891)	0.832 (0.769, 0.901)
Refined Grains	1.000	0.952 (0.884, 1.025)	0.872 (0.808, 0.941)	0.838 (0.776, 0.905)	0.823 (0.762, 0.890)
Sodium	1.000	0.908 (0.842, 0.979)	0.861 (0.798, 0.929)	0.849 (0.787, 0.917)	0.830 (0.769, 0.897)
Added Sugars	1.000	0.959 (0.889, 1.035)	0.902 (0.835, 0.974)	0.861 (0.796, 0.930)	0.839 (0.775, 0.908)
Saturated Fats	1.000	0.958 (0.888, 1.034)	0.933 (0.864, 1.008)	0.841 (0.777, 0.910)	0.858 (0.793, 0.929)

[1] *P*-trend < 0.0001 for all models. [2] Adjusted for self-reported covariates; age at study entry, body mass index, history of diabetes, energy, ethnicity, education, marital status, smoking, weekly hours of moderate to vigorous physical activity, and alcohol intake. [3] Adjusted for self-reported covariates; age at study entry, body mass index, history of diabetes, energy, ethnicity, education, marital status, smoking, weekly hours of moderate to vigorous physical activity, hormone replacement therapy, and alcohol intake.

Table S4. Hazard ratios (HR) (95% confidence intervals (CI)) for all-cause, cardiovascular disease (CVD), and cancer mortality according to quintiles of Healthy Eating Index-2015 (HEI-2015) scores in men (n = 70,170) stratified by ethnicity in the Multiethnic Cohort [1].

HEI-2015 Category	*n*	Any deaths *n*	Person-years of follow-up	All-cause mortality [1] HR (95% CI)	CVD deaths *n*	CVD mortality [1] HR (95% CI)	Cancer deaths *n*	Cancer mortality [1] HR (95% CI)
Men [1,2]								
White								
Quintile 1	2573	979	45,703	1.00 [3]	322	1.00 [3]	293	1.00 [4]
Quintile 2	2833	1033	50,771	0.88 (0.81, 0.96)	337	0.86 (0.73, 1.00)	320	0.94 (0.80, 1.11)
Quintile 3	3113	1093	56,951	0.80 (0.73, 0.87)	376	0.80 (0.69, 0.94)	331	0.87 (0.74, 1.02)
Quintile 4	3953	1336	72,705	0.76 (0.69, 0.82)	451	0.74 (0.64, 0.85)	421	0.86 (0.74, 1.01)
Quintile 5	4858	1622	90,356	0.69 (0.64, 0.75)	527	0.65 (0.56, 0.75)	475	0.76 (0.66, 0.89)
African American								
Quintile 1	1364	751	21,781	1.00 [3]	294	1.00 [3]	223	1.00 [5]
Quintile 2	1531	842	24,716	0.92 (0.83, 1.01)	305	0.82 (0.70, 0.96)	269	1.03 (0.86, 1.23)
Quintile 3	1734	917	28,177	0.88 (0.80, 0.97)	358	0.84 (0.72, 0.98)	268	0.94 (0.78, 1.13)
Quintile 4	1960	1003	32,351	0.82 (0.75, 0.91)	383	0.78 (0.67, 0.91)	313	0.94 (0.79, 1.12)
Quintile 5	2425	1198	41,436	0.71 (0.65, 0.79)	453	0.66 (0.57, 0.77)	343	0.79 (0.66, 0.94)
Native Hawaiian								
Quintile 1	1344	485	23,921	1.00 [6]	186	1.00 [6]	154	1.00 [6]
Quintile 2	1041	372	18,638	0.89 (0.78, 1.03)	123	0.74 (0.59, 0.94)	121	1.00 (0.78, 1.27)
Quintile 3	934	344	16,480	0.93 (0.81, 1.07)	132	0.88 (0.70, 1.11)	104	1.00 (0.77, 1.30)
Quintile 4	858	348	14,913	0.94 (0.81, 1.08)	129	0.83 (0.66, 1.05)	99	1.02 (0.78, 1.33)
Quintile 5	815	350	14,276	0.98 (0.85, 1.13)	130	0.88 (0.69, 1.11)	95	1.03 (0.79, 1.35)
Japanese American								
Quintile 1	5113	1567	94,659	1.00 [3]	496	1.00 [4]	514	1.00 [5]
Quintile 2	4572	1598	83,057	0.96 (0.90, 1.03)	511	0.96 (0.85, 1.09)	482	0.96 (0.84, 1.08)
Quintile 3	4238	1549	76,372	0.87 (0.81, 0.94)	481	0.83 (0.73, 0.94)	458	0.90 (0.80, 1.03)
Quintile 4	3787	1447	68,387	0.84 (0.78, 0.90)	487	0.85 (0.75, 0.96)	406	0.87 (0.76, 1.00)
Quintile 5	3529	1455	63,441	0.82 (0.76, 0.89)	477	0.81 (0.71, 0.93)	372	0.83 (0.72, 0.95)
Latino								
Quintile 1	3640	1221	66,034	1.00 [5]	410	1.00 [6]	382	1.00 [5]
Quintile 2	4057	1332	73,331	0.95 (0.87, 1.02)	464	0.98 (0.85, 1.12)	406	0.94 (0.82, 1.09)
Quintile 3	4016	1418	72,093	0.94 (0.87, 1.01)	517	1.00 (0.87, 1.14)	411	0.92 (0.80, 1.06)
Quintile 4	3475	1221	62,618	0.89 (0.82, 0.97)	444	0.94 (0.82, 1.07)	322	0.81 (0.70, 0.95)
Quintile 5	2407	895	42,981	0.88 (0.80, 0.96)	337	0.95 (0.81, 1.10)	230	0.79 (0.67, 0.94)

[1] Adjusted for self-reported covariates; age at study entry, body mass index, history of diabetes, energy, education, marital status, smoking, weekly hours of moderate to vigorous physical activity, and alcohol intake. [2] Quintile 1 is lowest score and quintile 5 is highest score, HEI ranges shown in Table 2. [3] p for trend <0.0001, [4] p for trend <0.001, [5] p for trend <0.01, [6] p for trend not significant.

Table S5. Hazard ratios (HR) (95% confidence intervals (CI)) for all-cause, cardiovascular disease (CVD), and cancer mortality according to quintiles of Healthy Eating Index-2015 (HEI-2015) scores in women (n = 86,634) stratified by ethnicity in the Multiethnic Cohort [1].

HEI-2015 Category	*n*	Any deaths *n*	Person-years of follow-up	All-cause mortality [1] HR (95% CI)	CVD deaths *n*	CVD mortality [1] HR (95% CI)	Cancer deaths *n*	Cancer mortality [1] HR (95% CI)
Women [1,2]								
White								
Quintile 1	3270	996	60,739	1.00 [3]	276	1.00 [3]	307	1.00 [3]
Quintile 2	3430	964	64,502	0.85 (0.78, 0.93)	285	0.87 (0.74, 1.03)	280	0.85 (0.72, 1.00)
Quintile 3	4066	1128	76,961	0.77 (0.71, 0.84)	379	0.87 (0.75, 1.02)	332	0.82 (0.70, 0.96)
Quintile 4	4729	1269	90,655	0.71 (0.65, 0.78)	368	0.68 (0.58, 0.80)	356	0.74 (0.63, 0.87)
Quintile 5	5158	1466	98,501	0.66 (0.60, 0.72)	438	0.63 (0.54, 0.74)	406	0.71 (0.61, 0.84)
African American								
Quintile 1	2412	980	42,472	1.00 [3]	383	1.00 [3]	270	1.00 [4]
Quintile 2	2720	1098	48,874	0.89 (0.81, 0.97)	454	0.92 (0.80, 1.05)	295	0.92 (0.78, 1.09)
Quintile 3	3108	1272	55,071	0.85 (0.78, 0.93)	493	0.81 (0.71, 0.93)	359	0.97 (0.83, 1.14)
Quintile 4	3578	1476	64,254	0.82 (0.75, 0.89)	627	0.86 (0.75, 0.98)	375	0.88 (0.75, 1.03)
Quintile 5	4254	1681	77,482	0.72 (0.67, 0.79)	640	0.67 (0.59, 0.77)	474	0.90 (0.77, 1.05)
Native Hawaiian								
Quintile 1	1662	435	31,071	1.00 [4]	132	1.00 [4]	147	1.00 [4]
Quintile 2	1324	368	24,625	0.96 (0.83, 1.11)	123	0.97 (0.75, 1.25)	110	0.94 (0.73, 1.21)
Quintile 3	1199	345	22,394	0.88 (0.76, 1.01)	110	0.85 (0.66, 1.11)	98	0.86 (0.66, 1.12)
Quintile 4	1134	337	21,034	0.85 (0.73, 0.99)	124	0.93 (0.72, 1.21)	88	0.81 (0.61, 1.07)
Quintile 5	1049	376	19,150	0.97 (0.83, 1.12)	126	0.95 (0.73, 1.23)	104	0.99 (0.75, 1.30)
Japanese American								
Quintile 1	5227	1086	102,263	1.00 [5]	326	1.00 [6]	338	1.00 [4]
Quintile 2	5335	1264	103,320	0.95 (0.88, 1.04)	422	1.02 (0.88, 1.18)	344	0.94 (0.81, 1.10)
Quintile 3	4984	1291	95,993	0.93 (0.85, 1.01)	428	0.95 (0.82, 1.10)	346	0.96 (0.83, 1.13)
Quintile 4	4697	1258	90,270	0.87 (0.80, 0.94)	400	0.84 (0.72, 0.98)	310	0.87 (0.74, 1.02)
Quintile 5	4542	1338	86,923	0.87 (0.80, 0.95)	427	0.84 (0.73, 0.98)	321	0.90 (0.76, 1.06)
Latina								
Quintile 1	4756	1106	91,007	1.00 [6]	376	1.00 [4]	336	1.00 [7]
Quintile 2	4517	1115	86,042	0.95 (0.87, 1.03)	353	0.86 (0.74, 1.00)	336	1.01 (0.86, 1.17)
Quintile 3	3971	984	75,519	0.89 (0.82, 0.98)	337	0.86 (0.73, 1.00)	250	0.82 (0.70, 0.97)
Quintile 4	3189	779	60,590	0.85 (0.77, 0.93)	263	0.82 (0.70, 0.97)	224	0.89 (0.75, 1.06)
Quintile 5	2323	654	43,781	0.92 (0.83, 1.02)	242	0.96 (0.81, 1.14)	160	0.83 (0.68, 1.01)

[1]Adjusted for self-reported covariates; age at study entry, body mass index, history of diabetes, energy, education, marital status, smoking, physical activity, hormone replacement therapy, and alcohol intake.[2]Quintile 1 is lowest score and quintile 5 is highest score, HEI ranges shown in Table 2. [3] p for trend <0.0001, [4] p for trend not significant, [5] p for trend <0.001, [6] p for trend <0.01, [7] p for trend <0.05.
